# A Re-Evaluation of the Size of the White Shark (*Carcharodon carcharias*) Population off California, USA

**DOI:** 10.1371/journal.pone.0098078

**Published:** 2014-06-16

**Authors:** George H. Burgess, Barry D. Bruce, Gregor M. Cailliet, Kenneth J. Goldman, R. Dean Grubbs, Christopher G. Lowe, M. Aaron MacNeil, Henry F. Mollet, Kevin C. Weng, John B. O'Sullivan

**Affiliations:** 1 Florida Program for Shark Research, Florida Museum of Natural History, University of Florida, Gainesville, Florida, United States of America; 2 Commonwealth Scientific and Industrial Research Organization Wealth from Oceans Flagship, Marine and Atmospheric Research, Hobart, TAS, Australia; 3 Moss Landing Marine Laboratory, Moss Landing, California, United States of America; 4 Alaska Department of Fish and Game, Homer, Alaska, United States of America; 5 Florida State University Coastal and Marine Laboratory, St. Teresa, Florida, United States of America; 6 Department of Biological Sciences, California State University Long Beach, Long Beach, California, United States of America; 7 Australian Institute of Marine Science, Townsville, QLD, Australia; 8 Pelagic Fisheries Research Program, Department of Oceanography, University of Hawaii at Manoa, Honolulu, Hawaii, United States of America; 9 Monterey Bay Aquarium, Monterey, California, United States of America; The University of Adelaide, Australia

## Abstract

White sharks are highly migratory and segregate by sex, age and size. Unlike marine mammals, they neither surface to breathe nor frequent haul-out sites, hindering generation of abundance data required to estimate population size. A recent tag-recapture study used photographic identifications of white sharks at two aggregation sites to estimate abundance in “central California” at 219 mature and sub-adult individuals. They concluded this represented approximately one-half of the total abundance of mature and sub-adult sharks in the entire eastern North Pacific Ocean (ENP). This low estimate generated great concern within the conservation community, prompting petitions for governmental endangered species designations. We critically examine that study and find violations of model assumptions that, when considered in total, lead to population underestimates. We also use a Bayesian mixture model to demonstrate that the inclusion of transient sharks, characteristic of white shark aggregation sites, would substantially increase abundance estimates for the adults and sub-adults in the surveyed sub-population. Using a dataset obtained from the same sampling locations and widely accepted demographic methodology, our analysis indicates a minimum all-life stages population size of >2000 individuals in the California subpopulation is required to account for the number and size range of individual sharks observed at the two sampled sites. Even accounting for methodological and conceptual biases, an extrapolation of these data to estimate the white shark population size throughout the ENP is inappropriate. The true ENP white shark population size is likely several-fold greater as both our study and the original published estimate exclude non-aggregating sharks and those that independently aggregate at other important ENP sites. Accurately estimating the central California and ENP white shark population size requires methodologies that account for biases introduced by sampling a limited number of sites and that account for all life history stages across the species' range of habitats.

## Introduction

Apex predators play an important role in marine community structure through both consumptive and non-consumptive effects on prey [1], [2], [3], [4]. Within marine systems, several species of large sharks are thought to be apex predators that may act as keystone species [2], [5]. Owing to their life history characteristics, sharks are thought to be particularly sensitive to overfishing and although there is much debate as to the extent, there is evidence of substantial declines in some locations (e.g. [6], [7], but see [8], [9], [10]).

The white shark (*Carcharodon carcharias*) is one of the largest and most widely known shark species, is cosmopolitan in distribution, and is known to aggregate off the coasts of California, Baja California Mexico, South Africa, New Zealand and southern Australia. While some life history aspects are poorly quantified, their diet [11], [12], [13], [14], local and long-distance movements [15], [16], [17], [18], [19], [20] as well as residency patterns at aggregation sites [45],[23],[39],[42] have been relatively well documented. Due to the vulnerability inferred from their longevity, growth rate and low fecundity, combined with perceived population declines and concerns for the potential of overfishing (through commercial bycatch and targeted fishing), white sharks have been protected under legislation or fisheries restrictions in South Africa (1991), Australia (1999), Mexico (2002), New Zealand (2007) and areas of the United States (California in 1994; northwest Atlantic and Gulf of Mexico in 1997; remainder of U.S. EEZ in 2005). While this affords a measure of protection in these areas, white sharks range widely across jurisdictional boundaries, making them susceptible to oceanic and coastal fisheries outside these areas. These concerns have led to listings through the Convention on International Trade in Endangered Species of Wild Fauna and Flora (CITES), the Convention on Migratory Species (CMS), and the International Union for Conservation of Nature (IUCN), as well as international efforts to estimate life history and demographic parameters, and quantify population sizes and dynamics.

Quantifying population parameters for white sharks is challenging because of their wide-ranging movements and naturally low numbers at any given location relative to lower trophic level predators and prey. Various methods have been proposed to estimate population sizes and track abundance trajectories [21]. A recent study by Chapple *et al*. [22] applied one of those methods, tag-recapture modeling, to photographic identifications of white shark individuals seasonally aggregating around two nearby pinniped rookeries (Farallon Islands and Tomales Point) to estimate the population size of white sharks off the central California coast, USA. The authors used the results from a Bayesian hypergeometric model to propose that the central California population consists of only 219 mature and sub-adult white sharks (highest-posterior density estimate) with a 95% credible interval of 130 and 275, where sub-adults are defined as being sexually immature, older juveniles found with adults in aggregation areas; *sensu stricto*: [23]). Chapple *et al* [22] further concluded that this represented ∼50% of the mature and sub-adult individuals for the entire eastern North Pacific Ocean (ENP), and that, when all life stages were considered, the total population of white sharks was “far lower” than that of other apex predators.

This very low estimate has created serious concern about the status of the California white shark population, and consequently resulted in petitions to consider white sharks as a candidate for the U.S. (Federal Register/Vol. 77, No. 189/Friday, September 28, 2012/Proposed Rules) and State of California endangered species lists. Under the Federal Endangered Species Act, an endangered species is defined as “any species which is in danger of extinction throughout all or a significant portion of its range.” As limited resources are best used in protecting species that are truly at risk of extinction, unwarranted petitions may draw resources away from more deserving species.

Chapple *et al*. [22] compared their estimate of white shark abundance in the ENP to geographically disparate sub-populations of two marine mammal apex predators, the killer whale (*Orcinus orca*: 1145 individuals in the ENP, Gulf of Alaska and Aleutian Islands) and the polar bear (*Ursus maritimus*: 1526 individuals in the Bering Sea), suggesting that healthy white shark populations should be of similar magnitude. Although the comparison to air breathing mammals may be inappropriate, the central California abundance estimate of Chapple *et al*. [22] for adults and sub-adults was much smaller than those in Australia [24], [25], [26] and South Africa [27], [28] (Table 1). The low estimates generated by Chapple *et al*. [22] are indeed alarming and, if accurate, justifiably raise concerns for the status of the population. However, they also require careful consideration for potential methodological flaws and assumption biases that may have resulted in underestimation of actual abundance. Successful conservation and management of this apex species demands that the scientific community report the most accurate information possible, with full disclosure of uncertainties and potential errors, lest limited resources and protective measures be misdirected.

**Table 1:** Population estimates and enumerations of white sharks from known aggregation areas around the world.

Source	Locale	Number of Individuals	Scale	Recaptured/Marked	Life Stages	Details
Cliff *et al*. [27]	Kwazulu-Natal to West Cape, South Africa	377–2,227 (1,279)	Regional	6/73	All	Mixed methods, JS assumptions met
Strong *et al*. [24]	Dangerous Reef/Spencer Gulf, South Australia	37–1612 (192)	Local	23/40	Adult and sub-adult	Mark Recapture, JS assumptions met
Martin [53]	Alaska, USA and British Columbia, Canada	29 (E)	Regional	n/a	Adult and sub-adult	Direct observation
Malcolm *et al*. [25]	South Australia	2,728–13,746	Regional to Continental	n/a	>1 year females	Deterministic minimum size model
Blower *et al*. [26]	Australia	1,512	Continental	n/a	Adult	Genetic techniques; “effective population” size
Nasby-Lucas and Domeier [83]	Guadalupe Island, Mexico	142 (E)	Local	n/a	Adult and sub- adult	Photo-identification only
Sosa-Nishizaki *et al*. [54]	Guadalupe Island, Mexico	114–134 (120)	Local	101/113	Adult and sub-adult	Mark/Recapture with JS via Photo-identification
Jorgensen *et al*. [19]	Central California, USA	179 (E)	Local	n/a	Adult and sub-adult	Encounter data
Chapple [84]	Central California, USA	197–360(251)	Local	28/131	Adult and sub-adult	Dissertation data
Chapple *et al*. [22]	Central California, USA	130–450 (219)	Local	38/130	Adult and sub-adult	Mark/Recapture via Photo-identification
Chapple *et al*. [22]	North Eastern Pacific	<<1100	Regional	n/a	All	Extrapolation from local study
Lowe *et al*. [68]	Southern California, USA	112 (E)	Regional	n/a	Juvenile	Fisheries interactions since 2000
Towner *et al*. [28]	Gansbaai, South Africa	808–1008 (908)	Local	∼300/532	Not reported	Mark/Recapture with JS via Photo-identification
This study	Coastal California, USA	2,148–2,819 (2418)	Regional	n/a	All	Demographics using Jorgensen *et al*. [19] and Chapple *et al*. [22]

Estimates presented as 95% confidence/credible interval range (average or most probable number). Direct enumeration indicated by (E); JS indicates a Jolly-Seber based open population mark/recapture model was used; n/a  =  not applicable to study.

We therefore revisit Chapple *et al*.'s [22] hypergeometric model and assess the impact of their assumptions on the estimated sub-population size. We then evaluate the impact that including transient sharks, a feature of white shark aggregations worldwide, would have on abundance estimates using a simulated data set. Finally, we present a new minimum total population estimate for the white sharks off the California coast using demographics, life history matrix information, and a conservative extension of Chapple *et al*.'s [22] estimate to include age classes that were either poorly sampled, or not sampled at all, by their methodology. We do not estimate the total population of white sharks throughout the ENP as there are insufficient length, age and abundance data on which to base demographic models for the entire region.

## Assessment of the Assumptions for Hypergeometric Population Model Estimates

Chapple *et al*. [22] conducted a seasonal tag-recapture study, annually sampling four to five month periods (Sep-Jan) during portions of 2006–2008 at two of several known mature and sub-adult white shark aggregation sites in central California, the Farallon Islands and Tomales Point. Sampling was timed to coincide with periods when white sharks are known to aggregate at these localities. They lured white sharks to a vessel with decoys and bait, photographed their fins, and used natural markings and morphological patterns from the trailing edge of the first dorsal fin to visually identify individuals. We use the word “tag” to define these unique natural markings. They then manually searched photographs taken in subsequent surveys for previously identified (i.e. “tagged”) sharks, considering “re-sightings” as “recaptures.” These tag-recapture results were incorporated into eight different published population estimation models, including a Bayesian hypergeometric model that was ultimately selected to estimate sub-population size, for unexplained reasons. The chosen model had five key assumptions: (a) the population is closed; (b) there is homogeneous sampling of animals and all individuals have an equal probability of capture; (c) the “tagging” process does not influence the chance of recapture; (d) there is zero “tag” loss; and (e) the photo-identifications of sharks at these two aggregation sites represent a random sample of the central California population. Implicit in the above assumptions is that all mature and sub-adult individuals in the central California population would have an equal chance of visiting these two aggregation sites each year during the three-year period of the study. Herein we evaluate each of these assumptions and their impacts on estimation bias.

### Closed population

Closed population models fundamentally assume that a population under study does not include birth, death, immigration or emigration over the period of sampling. Although violated routinely in practice, provided these changes occur randomly, only a loss of precision should result, with estimates remaining unbiased [29]. Chapple *et al*.'s [22] study violates the closure assumption in several important and non-random ways.

Satellite tagging of white sharks at aggregation sites off central California has revealed clear seasonal movement patterns, with sharks present near coastal and insular pinniped rookeries during the fall and swimming to an offshore focal zone or Hawaii in the spring and summer [18], [19], [30]. Of the sharks acoustically tagged in one of these studies, only 13 of 51 showed evidence of annual return to photographic sampling sites in central California. Only three sharks showed evidence of return each year for the three years of the study (2006–2008), although this figure no doubt underestimates the frequency of annual returns as not all sharks were tagged in year one ([19], fig. 5a). Yet Chapple *et al*. [22] concluded that all mature and sub-adult white sharks return annually to these pinniped rookeries. While lack of detection does not prove absence and externally placed tags are known to shed from sharks, no convincing evidence for an annual return cycle of all sharks was provided, and none is available in the literature.

Jorgensen *et al*. [19] acoustically detected sharks for up to 766 days, but most tracking durations spanned less than one annual cycle (mean 199±97 days). Female white sharks have been hypothesized to have a gestation period in excess of 12 months [31], which is supported demographically [32] and by observations of the biennial presence of females at the Farallon Islands [33]. Thus, mature females need to be tracked for at least two years to characterize their migration cycles. A recent study of female white sharks in the ENP presented satellite acquired tracks of two-year durations in the open ocean [20]. Furthermore, a compilation of observations and records for Hawaii showed that white sharks can be recorded there throughout the year [34], meaning that if they are part of the central California sub-population, not all individuals return to coastal aggregation areas during the northern autumn each year. Similarly, a compilation of records for Alaska and British Columbia reveals that white sharks occur there during Autumn-Winter periods when other animals are aggregated at California seal rookeries (53), indicating that such animals would not be accounted for by Chapple et al's methods.

Long-term photo identification of five individual sharks at the Farallon Islands from 1987 to 2008 showed that all individuals were absent from data records for periods of 1–9 yrs [35] with none showing a consistent pattern of periodicity between re-sighting, indicating that either those individuals did not visit annually or that they visited but were not re-sighted. The number of unique individuals identified by Chapple *et al*. [22] increased each year (41 in 2006, 42 in 2007 and 47 in 2008), suggesting an immigration of sharks during the study (including the possible appearance of transient sharks). Direct evidence also exists of individuals that were initially recorded at the Farallon Islands, then were later re-sighted much farther south, precluding annual returns to the Farallon Islands in those northern autumn seasons. For example, an individual white shark was photographed at the Farallon Islands during the fall of 1988, never re-sighted there, and subsequently re-sighted off of southern California during the fall six years later [36]. The shark either did not return to the Farallon Islands again or was never re-sighted if it did. Another white shark photographed in the Farallon Islands in the fall of 1991 was re-sighted at Tomales Point in the fall of 1994, but was never re-sighted thereafter in the Farallon Islands or at Tomales Point [36]. Additionally, a female white shark was tagged with an acoustic tag at the Farallon Islands in 2008 and detected at Guadalupe Island, Mexico in 2009 (but not at the Farallon Islands [37]). The existing literature and Chapple *et al*.'s [22] own data therefore demonstrate that the central California sub-population was open over the three sampled periods, thus violating a key modeling assumption. The combined evidence makes a strong case against using a closed population structure in future analyses of white sharks in this region.

### Homogeneous sampling of individuals

Chapple *et al*. [22] sampled two aggregation sites, assuming that every shark had an equal chance of being sighted and that sharks at the two aggregation sites mix homogenously with each other. While white sharks do show site fidelity to pinniped aggregation sites [17], [19], [34], [38], they have shown preference to specific islands and may restrict movements and thus limit mixing over small spatial scales. In Australian waters only 52% of white sharks tagged off the North Neptune Islands visited a pinniped aggregation site only 12 km away at the South Neptune Islands [39]. In the central California region, white sharks also show fidelity to particular aggregation sites including the sites that Chapple *et al*. [22] sampled; in a previous study using acoustic tags [19] sharks showed a strong propensity to reside at and revisit the site of tagging with relatively little exchange between four aggregation sites including Tomales Point and the Farallon Islands. If a shark was detected at another site, that detection period was short, suggesting that residents at one aggregation site might, at best, represent transient visitors at another. Thus, available data in the central Californian region, and more broadly for white sharks in general, indicate that they cannot be assumed to mix homogeneously over the spatial scale of the Chapple *et al*. study.

Therefore, based on their own data, Chapple *et al*. [22] should not have assumed homogeneous sampling. They attempted to account for heterogeneity by utilizing the Chao model [40], which resulted in a nearly 50% increase in the estimated population size (328 individuals) over the hypergeometric model. They also accounted for heterogeneity using the M_h_ model of Otis *et al*. [41]; however, this model uses a jack-knife estimator that assumes a fixed time-series of capture occasions, unlikely to be the case over the four to five month sampling period each year. The results of these analyses and the same acoustic tag data set available for their study demonstrate that the assumption of homogeneous sampling by Chapple *et al*. [22] was violated. This results in a low bias to their population estimate as their methods provide an increased probability of resampling previously tagged sharks.

### Tagging method does not affect subsequent chance of sampling

The technique employed by Chapple *et al*. [22], luring and baiting sharks to the surface where they can be photographed, assumes that all sharks have an equal chance of being attracted and that once photographed, all sharks in the area will have an equal chance of being attracted again. Delaney *et al*. [42] compared acoustic detections and photo-identification for evaluating residency times of white sharks in Mossel Bay, South Africa, and found photo-identification had the lowest probability of detection and underestimated residency (and local abundance). Additionally, there are often dominance patterns in which subordinate (smaller) white sharks are excluded from the area and from approaching the surface, or relegated to the visual periphery, leading to a greater probability of low quality photographs [24], [43], [44], and under-reporting (this also contradicts the aforementioned homogeneous sampling assumption). Finally, negative attraction conditioning, whereby “trap-shy” sharks learn that the bait and decoy do not lead to a food reward, diminishes the probability that they will be attracted during subsequent sampling events over time [45]. The same phenomenon has been observed at the Farallon Islands where white sharks have been observed approaching a floating video decoy (decoy with a video camera aimed under water) without surfacing, i.e., the observer(s) would not have detected the presence of a shark without the benefit of such underwater video footage (K.J. Goldman and S.D. Anderson pers. obs.). Failure to detect previously marked individuals would cause population size to be overestimated, whereas both the failure to detect unmarked individuals and the effect of dominance behavior would cause population size to be underestimated. While the net effect on estimation bias of not detecting marked vs. unmarked individuals cannot be fully evaluated, the dominance effects created by using bait and decoy attraction methods would, by themselves, effectively cause negative bias leading to an underestimation of population size. These observations suggest that the attraction, photo-identification and monitoring methods used by Chapple *et al*. [22] underestimate both local abundance and population size.

### Zero tag loss

Chapple *et al*. [22] utilized markings on the trailing edge of the dorsal fin to uniquely “tag” each individual shark. While this method can in some cases provide long-term identification, unlike a fingerprint, the pattern may change over time [46], [47], and marking changes leading to ‘tag loss’ (i.e. change in trailing edge of dorsal fin) could lead to over-estimation of the population. However, tag-loss does not necessarily result in overestimation as its effects have been shown to be dependent on capture probability in certain models [48]. A combination of genetics and photo-identification revealed that the method was 85% accurate at identifying individuals over a five year period in Mossel Bay in South Africa [49], indicating that a substantial proportion of individuals can be present but not positively identified. To that end, Chapple *et al*. [22] state that low quality photographs were discarded and, while they provided details in their supplemental information on how photographs were processed and eliminated, they offered no indication of the frequency of photograph rejections.

### Random sampling the central California sub-population using photo identification

The most serious violation of the assumptions of the models employed by Chapple *et al*. [22] is that the sharks they sampled at the Farallon Islands and Tomales Point represent random samples of the central California white shark sub-population. In fact, the sharks sampled at these locations represent only sharks that visit these two aggregation sites (either a single time or consistently over the years of their study). This conclusion is supported by the acoustic tagging study of Jorgensen *et al*. [19] who demonstrate that sharks largely reside at one aggregation site and less regularly visit other sites in the central Californian region, if at all. Therefore, the Chapple *et al*. study represents only a sub-sample of central California as they did not sample elsewhere, particularly other known white shark aggregation sites in the region, notably Año Nuevo Island [11], [50], which they excluded from their modelling for “logistical reasons.”

White sharks are known to enter and leave the sampled aggregation sites during the time that the photo-identification study was conducted, and hence they would not be available for sampling during the purportedly closed sampling periods [18], [19], [51]. All white sharks appear to seasonally exit Farallon Islands waters during the winter and spring, yet sharks are still seen along the mainland coastline during these periods. Year-round predation events on marine mammals, including pinnipeds and sea otters, occur as far south as southern California [11] and white sharks are observed throughout the year in the coastal waters of California and Mexico [11], [52], and in Hawaii [33], and as far north as British Columbia and Alaska [53]. This strongly indicates that some white sharks do not regularly associate with specific aggregation sites and these individuals would not be included in any such population estimate. Similar photo-identification methods have been used to estimate the population size of white sharks at Guadalupe Island, Mexico over a nine year period [54]. Despite the much longer sampling period and fact that an open population model was included, the authors appropriately cautioned against using the estimate as anything other than the an index of the population size at Guadalupe Island, the largest known white shark aggregation site in Mexico.

Recent stable isotope analyses also suggest that some white sharks do not specialize on mammals but instead forage mostly on bony fishes and perhaps squid [12], [13], [14], [55], especially when offshore. The importance of fishes in the white shark diet has been noted throughout its range [56], [57], [58], [59]. Individuals preferring piscine prey are unlikely to be attracted to seal aggregation sites on a consistent, predictable basis, if at all.

Several factors we discuss above could have a positive bias resulting in an overestimation of population size (e.g. underestimation of the number of marked animals, failure to detect marked animals and, in some cases, tag loss), while several other factors could lead to a negative bias resulting in an underestimation of population size (e.g. underestimation of the number of unmarked animals, dominance behavior and animals not attracted to the bait site). While the net effect of certain biases cannot be determined, our detailed analysis of the assumptions required of the mark recapture model chosen by Chapple *et al*. [22] demonstrate that their results are biased low. The violations of homogeneous and random sampling along with the dominance behavior effect and large potential for animals to not be attracted to their study site (i.e. baiting station) outweigh potential positive biases. Furthermore, we have shown that their results are not representative of all mature and sub-adult white sharks off central California, rather only of those that annually or biennially frequented the two adjacent pinniped rookeries sampled during the 2006–2008 time period.

We now evaluate the potential numerical effects of the biases associated with the differential residency patterns apparent at those two aggregation sites.

## Hypergeometric Model Bias and the Impact of Transients

Having evaluated the hypergeometric model and assumptions used by Chapple *et al*. [22], we now employ a more appropriate model to simulate the residency patterns of the white sharks represented in the dataset used in the above study (i.e., those animals that visit the Farallon Islands and Tomales Point at larger size classes). Mark-recapture methods for large migratory marine animals have received considerable attention in recent years, a key insight of this work being that individuals sampled at local sites often represent sub-populations that are distinct components of a larger total population. In this way, shark populations sampled from fixed locations can be thought of as having three potential components: residents, including individuals frequently present at the sampling locations; transients, including individuals that are infrequently present at the sampling locations; and outsiders, including individuals that never use the sampling locations. The data presented by Jorgensen *et al*. [19] for white sharks tagged at Californian sites including the Farallon Islands and Tomales Point are consistent with these patterns of habitat use for white sharks in this region. Without auxiliary data, there is no information available regarding outsiders that do not visit the sampling locations. Therefore, the best one can do is estimate the portion of the sub-population consisting of residents and transients.

Fearnbach *et al*. [60] recently explored resident and transient population dynamics in a long-term mark-recapture study of dolphins using photo-resight data of a similar kind to the short-term study by Chapple *et al*. [22]. Their dynamic model included births and deaths, as well as a detection function that allowed for detection probabilities <1, properties well known to affect population estimates [61]. Critically, they estimated that the resident dolphin population in the northern Bahamas represented only 30% of the sub-population size, with considerably lower identification probabilities among transients than residents. By including births, deaths, and differential detection probabilities for both transient and resident groups, this approach provides a credible alternative to simple closed-population mark-recapture models for analysis of photo-resight data of highly-mobile marine species.

To illustrate the potential bias induced by non-random sampling, we compared the hypergeometric approach of Chapple *et al*. [22] to a population simulated under the Fearnbach *et al*. [60] model. Briefly, for each individual in our simulated population we generated a known population history, *x_i_*, indicating if the individual was alive across five simulated years:
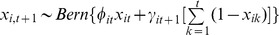
Thus, as defined by Fearnbach *et al*. [60], if an individual was alive at time t (i.e., *x_it_* = 1), then at time *t*+1 its status was given as a Bernoulli random variable with parameter *φ_it_*, the survival probability from time *t* to *t*+1. If an individual was not within the population during the previous time steps 1,…,*t* (i.e., *x_it_* = 0), then probability of entry into the population between intervals *t* and *t*+1 was a Bernoulli trial with parameter γ*_it_* = 1. For simplicity we assumed a stable population, fixing survival at φ*_it_* = 0.857 and entry into the sub-population, through birth, at γ*_it_*_+1_  = 0.153 [62]. Given these known fates of the population through time, we then generated sets of observed data through an observation process:
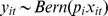
where *y_it_* is the identification history for shark *i* at time *t*, *p_i_* represents the probabilityof individual *i* being observed at any given time. These individual probabilities were simulated using conservative detection probabilities drawn from the time-averaged posterior resident and transient distributions of Fearnbach *et al*. [60]:
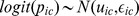
given approximate parent distributions

*u_r_* ∼ *N*(0.56,0.4) for residents

*u_t_* ∼ *N*(−1.7,1.6) for transients

across a range of sub-population mixtures, from all residents to mostly (90%) transients. By simulating across this range of population mixtures we were able to explore the effect of varying proportions of transients on estimates of population size. We consider the parent detection distributions to be conservative because marine mammals must surface to breathe and therefore are expected to be more frequently available for sampling than sharks that do not need to surface at all. Our approach included some probability of death and birth through time, which Chapple *et al*. [22] assumed did not occur during their study.

**Figure 1:**
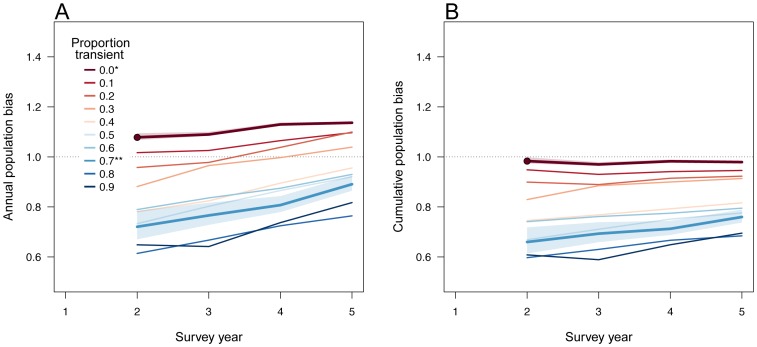
Relative bias in a closed-population hypergeometric model relative to a simulated dynamic sub-population. Results are shown for varying proportions of resident and transient individuals in a single simulated sub-population sampled from a fixed location (*sensu* Fearnbach *et al*. [60]); each combination of resident/transient is representative of repeated simulation runs showing: (A) Annual bias in a given survey year; (B) cumulative (i.e. discovery curve) bias. Values of 1 indicate equivalence (no bias) between methods; color variation represents proportion of known transients in the population from zero (dark red) to 90% (dark blue). *Assuming residents only from Chapple *et al*. [22]; **assuming resident/transient proportions from Fearnbach *et al*. [58].

Given the presence of both deaths and births in the simulated sub-population, two sources of hypergeometric bias become apparent through time. First, there are high and increasingly positive biases in the estimated number of individuals for sub-populations of residents only, even over two survey years (Figure 1a). This occurs because the model that generated the population includes births and deaths, while the hypergeometric model records only births. In other words, by only allowing for the accumulation of individuals in the population, the hypergeometric model leads to positive bias in some population estimates, even after only two sampling years, relative to the generating model that also includes some probability of death. How large this bias is initially depends largely on the proportion of transients in the population as their presence leads to downward biases in population size that become increasingly positive through time, again due to the accumulation of individuals recruited to the population that in the hypergeometric model will never die. Because the hypergeometric model retains all individuals ever observed in its estimate, the population closure assumption becomes increasingly tenuous through time. It also demonstrates that the proportion of transients vs. residents can have profound effects on population size.

Second, because the model cannot distinguish individuals, cumulative hypergeometric sub-population estimates (i.e. the total number of animals that have ever lived) are consistently low-biased, relative to the discovery curve for the known simulated population (Figure 1b). This reflects the fact that many transient individuals will go undetected over their lifetime, despite contributing to the total sub-population visiting the fixed sampling locations.

Our simulation results clearly show the large biases expected in a hypergeometric model given realistic population dynamics for a large, highly-mobile marine predator population. Failure to recognize that sub-populations sampled at a fixed locations include both resident and transient individuals generated downward population biases of between 22–33% in the first two years of sampling when 70% of individuals are transient [60]. These specific biases may be compounded by additional detection heterogeneity due to factors such as behaviour and the permanent emigration of individuals out of a given sub-population. However, even these more realistic model outputs cannot estimate the size of the total central California sub-population because they only contain information about the resident and transient individuals that visit fixed sampling locations, i.e. the sub-population. Estimating sub-population size over the broader central California area is outside of the scope provided by the data in both our and the Chapple *et al*. study. Such an estimate would require a stratified-random survey that sampled all insular and coastal aggregation sites as well as juvenile and other adult habitats, throughout the region. However, it is feasible to use the Chapple *et al*. data, accounting where possible for the biases discussed above, to estimate the minimum population required (all life history stages combined) to account for the estimated number of sharks that visit the Farallon Islands and Tomales Point sites.

We now address those calculations using demographic modelling.

## Demographic Population Model Estimates for All Age Classes

To extrapolate a population estimate for mature and sub-adult white sharks in central California to all life stages in the ENP, Chapple *et al*. [22] claimed that their estimate of 219 is “approximately half” of all mature and sub-adult ENP white sharks and erroneously cite two papers [63], [64] on juvenile white shark tagging experiments to draw conclusions on the abundance of white sharks younger than their sampling range. They then claim that the total population of white sharks in the ENP is “far lower” than that of other apex predators. Although we have demonstrated that their initial abundance estimate for mature and sub-adult white sharks in central California is biased low, we utilize this estimate along with the length distribution provided for the sharks sampled at the Farallon Islands and Tomales Point by Jorgensen *et al*. [19] to estimate how many sharks of all ages would be present if the Chapple *et al*. [22] mark-recapture based estimate and the Jorgensen *et al*. [19] length frequency data are accurate. While the results presented here unavoidably suffer from some of the low bias of Chapple *et al*.'s [22] original estimate (as we use their summary data), we consider them as useful *minimum* population sizes for all life stages of white sharks whose matures and sub-adults consistently visit these two locations. We do not extrapolate beyond this minimum number to estimate a total population size for white sharks in the ENP as data are lacking.

In extending Chapple *et al*.'s [22] sub-population estimate to produce a population estimate for all life stages of white sharks, we rely on well-established demographic methods based on previously published estimates of vital life history parameters [62]. We generated sex-specific size frequency tables for the 130 white sharks enumerated by Jorgensen *et al*. [19] assuming that these are the same sharks sampled by the parallel study of Chapple *et al*. [22], for which no data on individual sharks were provided. Lengths were converted to age using an established, although un-validated, length-age relationship [65]. An acoustic tag study [19] showed very low mixing of sharks between Año Nuevo Island and other aggregation sites (three of ten tagged sharks, only one with more than fleeting single detections); therefore, we specifically excluded the latter's Año Nuevo Island, California aggregation site data to more accurately utilize Chapple *et al*. 's [22] estimated 219 mature and sub-adult sharks.

We then used natural mortality and life history estimates based on an assumed longevity of 30 years and increased fertility to insure that lambda equaled 1.0, based on life history information in Mollet and Cailliet [62]. This allowed us to produce a survivorship curve and the expected age frequency distribution of white sharks of all life stages for this sub-population. We estimated the abundance of all life stages by matching the theoretical proportional age distribution to the realized age frequency of Jorgensen *et al*. [19], scaled to Chapple *et al.'s* [22] estimate, by matching the largest peaks in the sampled age frequency to the theoretical curve and applying the appropriate scaling factor to all other age classes.

Demographic analyses are a useful method for estimating population sizes and age distribution, and have been applied to several species of sharks, including white sharks [62], [66]. To do so, one needs a reasonable idea of age structure, often gained from size structure, and some age-specific population parameters. We discuss this approach and our analyses below.

### Age and size-based frequencies

**Figure 2:**
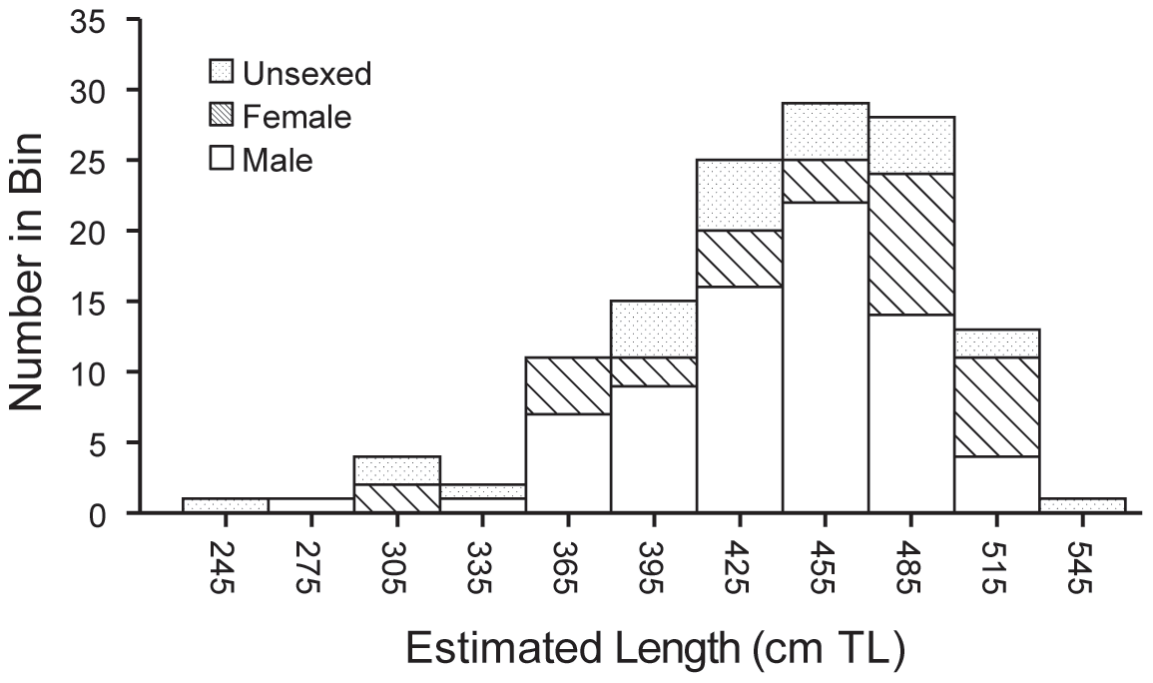
Size composition of white sharks enumerated near the Farallon Islands and Tomales Point, California, USA. Estimated total lengths ([TL] cm) of 130 white sharks (74 male, 32 female, and 24 unsexed) from Jorgensen et al. [19] were binned into 30 cm (∼1 ft) intervals. These data most likely represent the size and sex distribution of white sharks in Chapple *et al*.'s [22] mark-recapture population abundance estimates.

**Figure 3:**
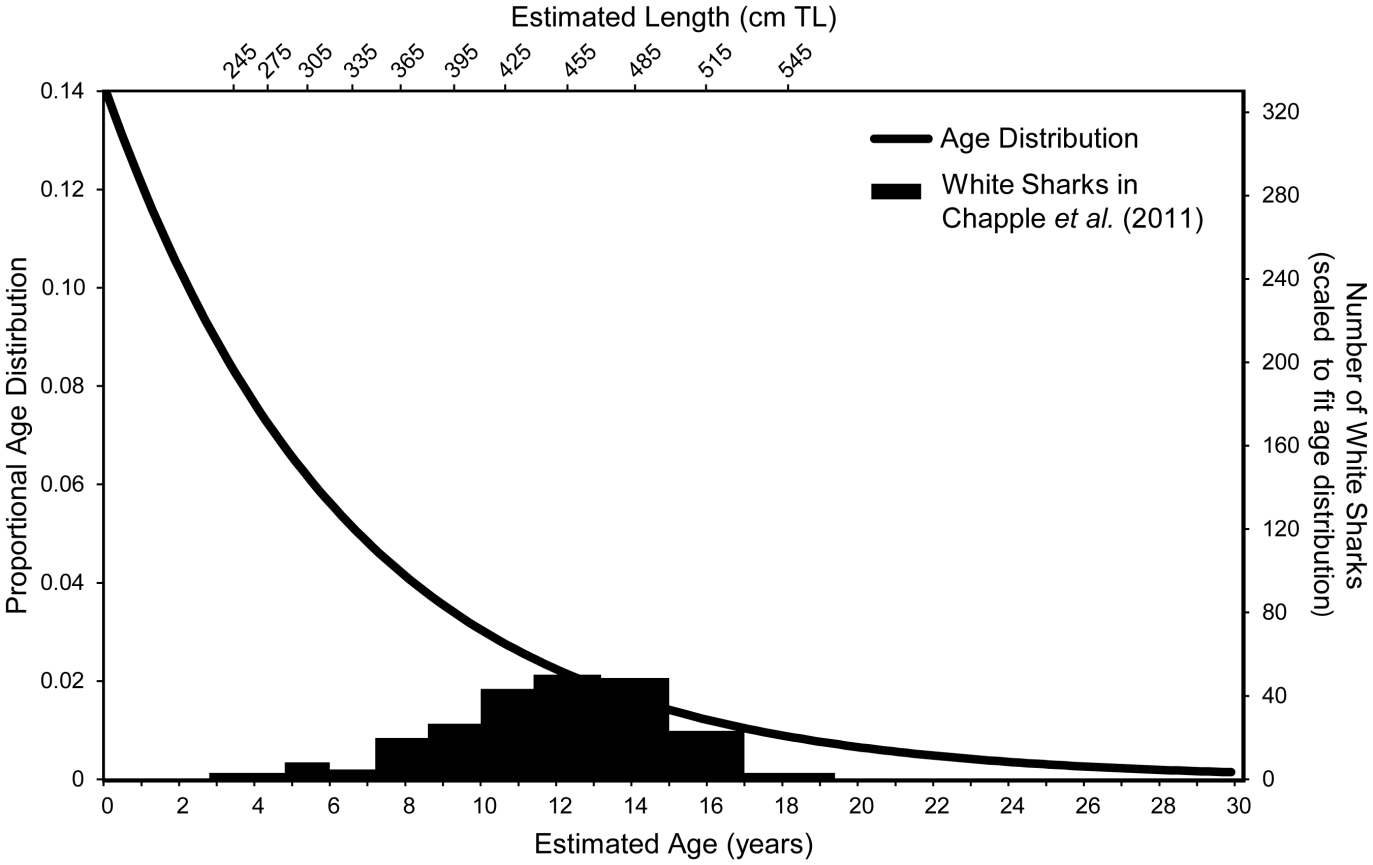
Age composition of white sharks estimated in mark-recapture study matched to the theoretical age distribution. The theoretical age distribution (black line with left y-axis) of all white sharks in coastal California, from birth to the predicted longevity of 30 years with a natural mortality coefficient (M) of 0.153 (∼86% survivorship), was determined using population parameters in Mollet and Cailliet [62]). The length-at-age relationship from Cailliet *et al*. [65] was used to determine the age of 130 white sharks (sexes combined) sampled by Jorgensen *et al*. [19], and presumably present in the Chapple *et al*. [22] study (see upper x-axis for lengths (TL cm)). The resulting age composition (black bars with right y-axis) was scaled to the mark-recapture population estimate and proportioned such that 100% of 13 year olds are accounted for in the Chapple *et al*. [22] population estimate of 219 white sharks.

The size-frequency and sex of the sharks sampled by Chapple *et al*. [22] can be inferred from Jorgensen *et al*. [19] as both studies were run in parallel (Figure 2). The estimated lengths of the 130 sharks enumerated by Jorgensen *et al*. [19] were binned at 30 cm intervals. These size and sex frequency data, when compared to estimated sizes at maturity (males ∼3.6 m, females at 4.5–5.0 m; [30], [67]), reveal that Jorgensen *et al*. [19] and Chapple *et al*. [22] primarily sampled mature males plus a few sub-adults and small mature females (sex ratio: 78 males:33 females, χ2 = 10.6176, p<0.01). Large mature females were almost entirely absent from their sub-population estimate, and other size classes were markedly under-represented (see Figures 2 and 3).

Chapple *et al*. [22] did not include juveniles in their Farallon Islands-Tomales Point estimate owing to rarity in their data set. This is not surprising because juvenile white sharks are known to inhabit inshore coastal habitats [39], [63], [64], [68], [69]. At pinniped rookeries, it is the larger individuals that most frequently attend seal kills or approach boats to investigate bait [24], [43], [44], indicating a likely bias towards more dominant, larger individuals in both photographic and electronic tagging records. Smaller individuals do feed at seal kills but are less frequently observed than larger individuals [44] even though, demographically, they should be more numerous in the total population. Smaller individuals thus are under-represented at seal kills either because they are present in lower numbers at seal colonies, or are excluded from kills by more dominant individuals, or both. As noted above, it is likely that individuals attracted, identified, or tagged in the Chapple *et al*. [22] study was inherently biased towards larger individuals.

The sex ratio of our sample data from Jorgensen *et al*. [19] is strongly male biased, indicating that females may occupy different habitats or locations. Sexual segregation by white sharks is common [70], [71], [72], [73]. Within False Bay, South Africa, females move into shallow water close to shore while males are predominantly found adjacent to islands [73]. Although based on relatively small sample sizes, observations of white sharks along the coastline of Point Reyes also suggest sex ratios favoring females close to shore [11].

### Demography

We used established demographic methodology [62], [74] to generate a survivorship curve using an assumed longevity of 30 years, a very conservative estimate given existing growth functions[65]; an instantaneous natural mortality estimate of M = 0.1535 (∼85.7% survivorship per year); age at maturity  = 15 years; and annual fertility (number of female pups per year)  = 1.553, a slightly higher rate than reported in the literature [27], [31], [32], [65], [62], [75] to achieve a stationary age distribution (lambda  = 1.0) [76], [77].

We then fit the size (age) frequency data for the 130 animals enumerated by Jorgensen *et al*. [19], scaled to reflect Chapple *et al*.'s [22] local population estimate of 219, to the theorized age distribution by assuming that the estimate of Chapple *et al*. [22] was correct for the most abundant age classes (Figure 3). By applying the proportion of estimated white sharks at a given age to those of all other age classes, we were able to derive a numerical estimate for all age classes combined.

Our results demonstrate that Chapple *et al*. [22] only sampled a small proportion of the age distribution for the sub-population represented at the sampling locations and that the total population of all life stages throughout coastal California would number at least 2,148 white sharks. We do not suspect this abundance to be “far lower” than that of other apex predators in the region. Matching the sampled age frequency of the most abundant age classes with neighboring age distribution proportions (at 12, 13 and 14 yrs) and scaling appropriately provides a range from 2,148 to 2,819 white sharks, with 2,418 as the more conservative value of two best fits at age 13 (Figure 3). Increasing M by a factor of two to reflect increased mortality of early life stages for each of the two youngest age classes results in a negligible effect (<2%) on the population estimate.

Recent length-at-age estimates of five male sharks from central California and eight sharks from the northwest Atlantic (four males, four females) suggest that growth rates may be much slower than our growth model predicts and that longevity may be much longer (up to 73 yrs for one male sampled) [35], [78]. If that is indeed the case, our age frequency/age distribution matching process would occur with sharks as much as twice as old and yield results ranging from ∼3450 to ∼5120 sharks to include additional younger and older sharks, even if we delay age at first maturity to a very conservative 30 yrs. Thus, our parameterizations for age, growth, longevity, mortality and fecundity yield quite conservative results with regards to the range of possible numerical values for vital parameters and model results. These initial calculations should motivate a more complete analysis that fully considers elasticities and uncertainties in salient vital parameter estimates to provide confidence bounded total population estimates that are beyond the scope of this paper.

Our demographic analysis, based on Chapple *et al*.'s [22] mark-recapture estimate for mature and sub-adult sharks at the Farallon Islands and Tomales Bay, suggests at least an order of magnitude more sharks comprise the total population when all life stages are accounted for. This approximation is in itself very conservative, as it does not include large numbers of mature and sub-adults missing in Chapple *et al*.'s [22] estimate, or those from sites other than the Farallon Islands and Tomales Point. Importantly, the skewed sex ratio and size frequency distribution reveal that a large proportion of the age classes for our estimated total population likely are not present or detected at either the Farallon Islands or Tomales Point and may indeed range throughout coastal California. As our demographic study also does not include known sharks at Año Nuevo, the true population size of white sharks throughout coastal California is likely to be significantly greater than our minimum population estimate of 2,418, and larger still if we were to estimate the population of all sharks in the ENP, including the significant numbers of all life stages of white sharks in Mexican waters.

## Discussion

Formal listing of a species as “endangered” places substantial demands on governments, which must devote considerable resources to protecting listed species, and on segments of the public, who must forgo social and economic opportunities to comply with applicable laws. Consideration and listing of species that are not under the threat of biological extinction may divert resources away from those that are genuinely at risk. It therefore is critical that the best possible information be used in listing reviews to minimize undue burdens and insure that limited resources are applied to recovery of the neediest species. Unfortunately there is considerable incentive for some conservation organizations to quickly support listing species, often after minimal critical scientific review, as listings can be cited as tangible achievements in the effort to preserve the environment and attain other conservation goals.

Chapple *et al*. [22] assumed their abundance estimate for sub-adult and mature white sharks in central California represented ∼50% of the sub-adult and mature population in the entire ENP. They compared their population estimate to those of marine mammals, including killer whales and polar bears, and this comparison was used as evidence that the ENP white shark population is smaller than should be expected. For example, Chapple *et al*. [22] misleadingly state that the entire population of killer whales in the ENP is 1,145, citing Barrett-Lennard and Ellis [79], who actually provided estimates for seven distinct sub-populations (ranging from 10 to 360 individuals) rather than an ENP population estimate. Chapple *et al*. [22] summed these seven estimates for a total of 1,145. The portion of the white shark population surveyed by Chapple *et al*. [22] is of comparable scale to one of these killer whale sub-populations, specifically the “West Coast Transient” sub-population (n = 219; [79]). Although our new estimate exceeds the estimated population size of both killer whales and polar bears reported by Chapple *et al*. [22], we consider such comparisons inappropriate.

In stating that the ENP white shark population is small relative to other apex predators such as killer whales and polar bears, Chapple *et al*. [22] implicitly assume that the ecology of each of these apex predators is similar and that the community structure, trophic interactions, and carrying capacities are also the same or similar. They are not. Additionally, since mark-recapture methods for estimating marine mammal population sizes are considered problematic, line-survey methods are typically employed for this purpose [80]. Such aircraft or ship based visual surveys have a high encounter rate as marine mammals must return to the surface to breathe; this is not the case for sharks [81]. This basic physiological difference, which leads to underestimates of shark population sizes in comparison with marine mammals, precludes direct comparisons of population estimates between the two groups.

Our new estimate is comparable to white shark population estimates from other locations and, importantly, our estimate is similar to that from a recent independent status review of the ENP white shark population, which estimated ∼3,000 individuals. The status review team had access to virtually all available data from white shark researchers throughout the eastern North Pacific and used multiple modeling approaches [82]. Previous studies in other locations used a variety of methods including mark/recapture, demographic analysis and genetic techniques over a range of different regions and locations (Table 1). One of the most comprehensive studies was conducted in Australia using catch and effort data, historical fishing records, shark control program records, cage-diving operation information, telemetry, tag and recapture, and shark attack data [25]. A deterministic population size model given the most probable population parameters was used to estimate a population size of between 2,728 and 13,746 female white sharks. Although the size of the central California coastline is significantly smaller than the area studied in Australia, the difference in population size estimates between this study [25] and Chapple *et al*. [22] is striking.

A mark-recapture study using photo-ID techniques at two adjacent white shark aggregations in Gaansbi, South Africa resulted in an abundance estimate of 908 (808–1008) sharks. This sub-population abundance is more than three times the Chapple et al. [22] mark-recapture estimate, in a region quite biogeographically similar to Farallon Islands and Tomales Bay [28]. This study employed open-population models and used a common quantitative technique (qAIC) to compare between competing model parameterizations, a feature absent from the analysis of Chapple et al. [22].

Genetic techniques were used to estimate the effective population size of white sharks off southern Australia at approximately 1,512 individuals [26]. The genetics also suggested that a minimum of 500–1,000 breeding individuals would be required to retain enough genetic variability and ensure evolutionary potential. Chapple *et al*. [22] also state that evidence of low genetic diversity in the ENP white shark population supports their assertion that the size of this population is alarmingly low, citing Jorgensen *et al*. [19]. Such low genetic diversity is common among shark populations even in populations that are healthy and unexploited (see [85] for review). Moreover, Jorgensen *et al*. [19] suggest the comparatively low genetic diversity in the ENP population of white sharks is due to its relatively recent establishment from a small number of founders, not the result of population decline.

Elasticity analyses have typically concluded that juvenile survival exerts the greatest influence on population growth rate of sharks in general [86] and that this is likely for white sharks in particular [62]. A recent study showed that incidental take of juvenile white sharks in southern California gill-net fisheries peaked in 1985, and CPUE declined until the mid-1990s. Extensive regulation of these gillnet fisheries in the mid-1990s resulted in substantial reduction and stabilization of effort and since then there has been a steady increase in the number of juveniles sharks reported caught, suggesting a recent population increase [68]. This suggests that existing measures to protect white sharks in the Californian region are achieving benefit and should continue to be monitored.

## Conclusions

Despite the many flaws outlined here, the Chapple *et al*. [22] estimate of 219 mature and sub-adult white sharks is useful as an index or *minimum* estimate for the numbers of predominantly male sharks that annually return to two pinniped rookeries in central California; we suspect, however the estimate of 328 (222–433) from the Chao model [40] to be more useful as it accounts for heterogeneity of capture probabilities. Extrapolation from any sub-population abundance estimate to include members of the population outside of the specific sex/age/length distributions or localities without accounting for sampling bias or including population demography may mislead or misinform conservation and management of this important apex predator. Our demographic results suggest that the white shark population abundance required to account for the number of mature and sub-adult sharks sighted by Chapple *et al*. [22] at the localities they surveyed must be at least an order of magnitude larger than their estimate when all age classes are included and is probably not “far lower” than other apex predators in coastal California or the ENP. Furthermore, there are signs that the California sub-population is at least stable with recent data suggesting increasing numbers of juveniles [68]. Our estimate and these recent observations place doubt on the need for inclusion of white sharks on the national or state endangered species lists and indicate that existing protective measures are likely to be improving the population status and should be maintained. More importantly, the status of the population should be regularly monitored using a variety of techniques, as no single method is likely to provide completely scientifically sound and unbiased results.
